# Bovine γδ T Cells Are a Major Regulatory T Cell Subset

**DOI:** 10.4049/jimmunol.1303398

**Published:** 2014-06-02

**Authors:** Efrain Guzman, Jayne Hope, Geraldine Taylor, Adrian L. Smith, Carolina Cubillos-Zapata, Bryan Charleston

**Affiliations:** * The Pirbright Institute, Surrey GU24 0NF, United Kingdom;; † The Roslin Institute University of Edinburgh, Midlothian EH259RG, United Kingdom; and; ‡ Department of Zoology, University of Oxford, Oxford OX1 3PS, United Kingdom

## Abstract

In humans and mice, γδ T cells represent <5% of the total circulating lymphocytes. In contrast, the γδ T cell compartment in ruminants accounts for 15–60% of the total circulating mononuclear lymphocytes. Despite the existence of CD4^+^CD25^high^ Foxp3^+^ T cells in the bovine system, these are neither anergic nor suppressive. We present evidence showing that bovine γδ T cells are the major regulatory T cell subset in peripheral blood. These γδ T cells spontaneously secrete IL-10 and proliferate in response to IL-10, TGF-β, and contact with APCs. IL-10–expressing γδ T cells inhibit Ag-specific and nonspecific proliferation of CD4^+^ and CD8^+^ T cells in vitro. APC subsets expressing IL-10 and TFG-β regulate proliferation of γδ T cells producing IL-10. We propose that γδ T cells are a major regulatory T cell population in the bovine system.

## Introduction

T cells expressing the γδ TCR have been described as nonclassical T cells, because unlike most TCR αβ T cells, activation can be independent of MHC–peptide complexes. In mice and humans, γδ T cells represent between 1 and 5% of the circulating lymphocytes, but are present at higher frequencies in epithelial sites (1). Many functions have been described for γδ T cells including cytokine production, Ag presentation, and immune regulation (2, 3). However, these various functions have been identified mostly for mice and humans, species with “low” numbers of circulating γδ T cells. In contrast, many other species such as cattle, sheep, pigs, and chickens are considered to have “high” numbers of circulating γδ T cells, and the function of these is yet to be determined. In the bovine system, γδ T cells represent between 15 and 60% of the circulating lymphocytes (4), and a large proportion of bovine γδ T cells express workshop cluster 1 (WC1), a transmembrane glycoprotein and member of the scavenger receptor cysteine-rich family, which is closely related to CD163. Although functional WC1 molecules have so far been identified only in ruminants, pigs, and camelids, WC1 orthologs have been identified in many other species (5).

Regulation of the immune system is important to prevent autoimmunity and immunopathology. Regulatory T cells (Tregs) are now recognized as a critical component of a balanced immune system (6, 7). The predominant Treg types are CD4^+^ and express either or both CD25 and the forkhead box transcription factor, Foxp3 (8). Despite the existence of bovine CD4^+^CD25^high^ Foxp3^+^ T cells, these cells have been shown to be neither anergic nor suppressive in vitro (9). Instead, mounting evidence supports the notion that γδ T cells are involved in immune suppression in ruminants. For example, depletion of γδ T cells from PBMC cultures resulted in increased Ag-specific proliferation and cytokine production in ex vivo cultures of T cells (10–12).

Tregs need to be licensed or activated to initiate and maintain their regulatory role. Dendritic cells (DCs) can prevent, inhibit, or modulate T cell–mediated responses through a variety of mechanisms ranging from the production of anti-inflammatory factors to the induction of T cell responses, which result in deletion, anergy, or instruction of regulatory cells. Immature DCs have been proposed to be tolerogenic (13), and this function is thought to be a consequence of the presentation of Ag in the absence of costimulation or cytokines. In addition, tolerogenicity of DC subsets may be dependent on the secretion of anti-inflammatory signals such as IL-10, TGF-β, and retinoic acid, among others (14).

In this report, we present evidence for the role of circulating γδ TCR^+^ cells as potent inhibitory T cells in the bovine system. Subsets of γδ T cells secreted IL-10 ex vivo and proliferated in response to IL-10, IL-4, and TGF-β, which, in turn, initiated a positive-feedback mechanism producing more IL-10 in proliferating γδ T cells. IL-10–expressing γδ T cells suppressed Ag-specific and nonspecific proliferation of CD4^+^ and CD8^+^ T cells. Suppressive γδ T cells were present in both WC1^+^ and WC1^−^ γδ TCR^+^ T cell populations, and were not stained with anti-Foxp3. We also identified specific subsets of APCs from various anatomical sites responsible for the expansion of γδ T cells with suppressive function and show that in vitro infection of APCs with modified vaccinia Ankara (MVA) increased the frequency of IL-10–expressing γδ T cells. These results suggest that a subset of circulating T cells expressing the γδ TCR are a major regulatory and suppressive T cell population in ruminants.

## Materials and Methods

### Animals

Conventionally reared Holstein cattle (*Bos taurus*) from The Pirbright Institute herd were used to obtain peripheral blood and tissues. All animals were at least 6 mo of age and from a bovine virus diarrhea virus (BVDV)–free herd. All animal experiments were approved by The Pirbright Institute ethics committee according to the U.K. Animal (Scientific Procedures) Act 1986.

### Vaccination and Ag-specific T cell assays

To obtain Ag-specific T cells, we vaccinated cattle (*n* = 10) with inactivated FMDV (foot-and-mouth disease virus) vaccine (O1 Manisa/A22 Iraq; Intervet, Milton Keynes, U.K.) as described previously (15). FMDV-specific proliferation, IFN-γ ELISPOT, and intracellular cytokine staining have all been described previously (15–17) using the FMDV vaccine Ag for Ag-specific stimulation. In some experiments, UV-inactivated BVDV was used as control Ag as described previously (18). In some assays, γδ T cells were removed by MACS as described later, and autologous γδ T cell subsets were added back to the starting cultures at a ratio of 1 γδ T cell to 1 PBMC.

### Separation and preparation of lymphocyte subsets

Heparinized venous blood was centrifuged at 300 × *g* over Histopaque 1083 (Sigma, Poole, U.K.), and the mononuclear cells were washed three times in PBS. Cells were either used immediately or frozen in FCS containing 10% DMSO (Sigma). CD14^+^ cells were purified by MACS using anti-human CD14^+^ microbeads (Miltenyi Biotec, Surrey, U.K.) (19). Monocyte-derived DCs (MoDCs) were prepared by culturing CD14^+^ cells for 5 d at 37°C in tissue culture media (RPMI 1640 [Invitrogen, Paisley, U.K.] containing 10% heat-inactivated FCS [Autogen Bioclear, U.K.], 2 mM l-glutamine, 55 μM 2-ME, penicillin [100 U/ml], and streptomycin [100 μg/ml]) and supplemented with recombinant bovine GM-CSF and IL-4 (The Pirbright Institute) (20). T cell subsets were positively selected from peripheral blood using MACS (Miltenyi Biotech) with the following Abs: CC58 (anti-CD8β) (21), CC8 (anti-CD4) (22), CC15 (anti-WC1) (23), and GB21A (anti-γδ TCR) (4) as described previously (17) and following the manufacturer’s instructions. In some assays, γδ T cells were negatively selected: PBMCs were depleted of MHC II^+^ cells (CC108, anti-MHC II) (24); CD8^+^ (CC58); CD4^+^ (CC8), B cells (CC21, anti-CD21; Serotec), and NK^+^ cells (AKS1, anti-NKp46) (25) using a MACS LD column (Miltenyi Biotec) following the manufacturer’s instructions. In all cases, the purities of the selected populations were analyzed by flow cytometry and were confirmed to be >98% pure.

### Lung-derived and afferent-lymph DCs

Lung-derived DCs were obtained as described previously (26, 27). A portion of lung tissue obtained postmortem was excised and weighed. Three grams of lung tissue was placed in a petri dish and chopped very finely with a pair of scissors and a scalpel blade. The sliced tissue was then placed in a 250-ml polycarbonate conical flask together with l00 ml RPMI 1640, 10% FCS containing 200 U/ml collagenase 4196 (Worthington) or hyaluronidase (BDH), and 50 U/ml DNase (Sigma). The tissue fragments were incubated for 2 h at 37°C in an orbital shaker (200 rpm). After digestion, the tissue was sheared through a 20-ml syringe, filtered through sterile gauze and muslin, and centrifuged for 10 min at 300 × *g* at 20°C. Mononuclear cells were separated on a Histopaque 1083 (Sigma) gradient as described earlier.

Afferent-lymph DCs (ALDCs) were obtained by surgical cannulation of the lymphatic vessels draining the suprascapular lymph node of cattle as described previously (28). Anti-bovine mAbs used to distinguish DCs from other cells were specific for MHC II (IL-A21), CD11c (IL-A16), DEC-205 (CC98), CD8α (CC63), and CD172a (CC149) (29). All Abs were obtained from The Pirbright Institute. DCs and subsets were flow sorted using a FACSAria II (Becton Dickson) as described previously (15).

### In vitro proliferation assays

Lymphocyte proliferation was measured by CFDA-SE (referred to as CFSE) dilution and analyzed by flow cytometry (16). To assess proliferation of γδ T cells in mixed cultures, we labeled PBMCs with CFSE (Invitrogen) following the manufacturer’s instructions and cultured them in tissue culture media for 5 d in 96-well U-bottom plates (Costar) at a concentration of 1 × 10^6^ cells/well in a total volume of 200 μl. In some cases, γδ T cells were positively or negatively selected, labeled with CFSE, and 5 × 10^5^ cells were cultured in 96-well U-bottom plates (Costar) with the same number of autologous irradiated (3000 rad) CD14^+^ cells in the presence of the following blocking Abs: CC320 (anti–IL-10) (30), CC313 (anti–IL-4) (31), CC328 (anti–TNF-α) (32), and anti–TGF-β (clone 1D11; R&D Systems), which have been previously shown to neutralize their target cytokine. In some cases, positively or negatively selected γδ T cells were cultured in plates coated with 10 μg/ml anti-bovine CD3 (clone MM1A; VMRD) and in the presence of 5 μg/ml anti-bovine CD28 (clone F849CD10; The Pirbright Institute) (33), 5 ng/ml rTGF-β1, and 50 U/ml human rIL-2 (Roche) as described previously (34). Polyclonal activation of T cells was performed using 20 ng/ml phorbol 12-myristate 13-acetate and 1 μg/ml ionomycin. For contact-dependent proliferation assays, 5 × 10^5^ irradiated CD14^+^ cells were added to the lower chamber of a 48-well Transwell plate (Costar); the 3-μm insert was carefully slotted in and autologous purified γδ T cells added to the upper chamber in the presence or absence of blocking Abs described earlier. In assays where γδ T cells were cultured with APC subsets, γδ T cells were positively or negatively selected, labeled with CFSE, and 5 × 10^5^ cells were cultured in 96-well U-bottom plates (Costar) with an equal number of autologous irradiated (3000 rad) FACS or MACS-sorted APCs (as described earlier) in a final volume of 250 μl. Proliferation and Ag presentation assays were performed as described earlier.

### Flow cytometry

Fluorochrome-labeled mouse anti-bovine mAbs used in this study have been described in detail previously (35–39). For cell-surface molecules, these were: CC149-PerCP/Cy5.5 (anti-SIRPα), ILA-16-AlexaFluor 680/PE (anti-CD11c), ILA-21-PE (anti-MHCII), ILA-156-PE/Texas Red (anti-CD40), CC30-allophycocyanin/Cy5.5 (anti-CD4), CC63-allophycocyanin/Cy7 (anti-CD8), IL-A29-allophycocyanin (anti-WC1), BAQ159A (anti-WC1.1; VMRD), CACTB32A (anti-WC1.2; VMRD), GB21a-AlexaFluor 405 (anti-γδ TCR; VMRD), and ILA-111-AlexaFluor 610/PE (anti-CD25). IL7Rα (CD127) was detected using an anti-mouse CD127 Ab raised in rats (clone RTK2758; Biolegend) and found to cross-react with bovine CD127 (this report). For intracellular staining, 10 μg/ml brefeldin A (Sigma) was added to cultured cells for 4 h and cells were harvested. Surface staining was performed in 96-well U-bottom plates (Costar) followed by fixation and permeabilization using BD Cytoperm/Cytofix kit following the manufacturer’s instructions. Intracellular cytokine staining was performed with CC302-PE or -AF488 (anti–IFN-γ), CC319-PE-Cy5.5 (anti–IL-10), CC312-PE/Texas Red (anti–IL-4), σG9-FITC (anti-perforin; BD Pharmingen), TW4-2F8 (anti–TGF-β1–PE/Cy7; Biolegend), FJK16s-PE (anti-mouse/rat Foxp3 that cross-reacts with cattle (40); eBioscience), and Ab86-allophycocyanin (anti–IL-2-AF657, a gift from A. Whelan, Animal Health and Veterinary Laboratories Agency, Weybridge, U.K.) (41). Control mAbs were isotype and concentration matched anti-turkey rhinotracheitis virus mAbs (28, 42). All Abs were either raised against bovine or confirmed to be cross-reactive with bovine cells and obtained from The Pirbright Institute except where noted. Dead cells were excluded using the 405-nm excitable dye Live/Dead Aqua or propidium iodide (Invitrogen) following the manufacturer’s instructions, and doublets were excluded using SSC-A/SSC-H. The cells were acquired using an LSRFortessa (Becton Dickinson), and staining was analyzed using FlowJo (TreeStar). Only live, single events were used for analysis, and at least 50,000 were used for postacquisition analysis. Proliferation analyses were performed using the Cellular Proliferation module on FCS Express v4 (DeNovo).

### ELISA

Bovine cytokine ELISAs were performed on culture supernatants as described previously for IL-10 (30), IL-12p40/70 (43), IL-4 (31), and IFN-γ (44). IL-2 was detected using the DuoSet Bovine IL-2 ELISA kit (R&D Systems) following the manufacturer’s instructions.

### Viruses

E1- and E3-deleted recombinant human adenovirus 5 (AdV5) and MVA expressing GFP were generated by the Jenner Institute Viral Vector Core Facility, University of Oxford, Oxford, U.K., and have been described previously (15). In some experiments, noncytopathic BVDV isolate PEC515 was used as described previously (18).

### Statistical analysis

Calculation of descriptive statistics (geometric statistics and SDs), nonparametric statistical analyses, and graphs were generated using GraphPad Prism for Windows v5.01 (GraphPad, San Diego, CA).

## Results

### γδ TCR^+^ T cells express IL-10 and have inhibitory function

**FIGURE 1.**
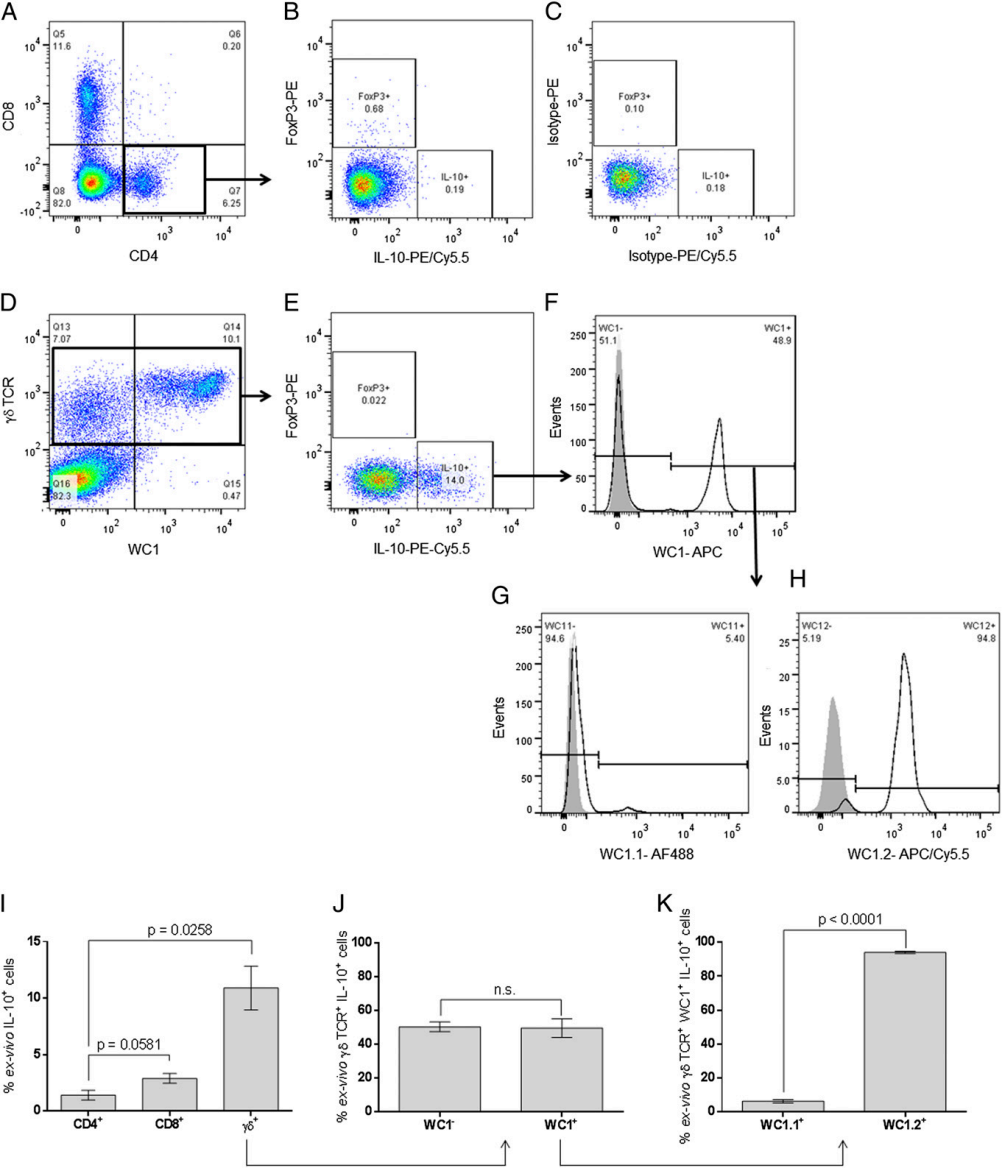
γδ T cells express IL-10 ex vivo. Whole blood was collected in heparin and incubated for 4 h at 37°C in the presence of brefeldin A (BFA). Cells were then fixed, permeabilized, stained, and analyzed by flow cytometry. (**A**) Frequencies of CD4^+^ and CD8^+^ T cells. (**B**) Expression of IL-10 and Foxp3 in the CD4^+^ population. (**C**) Isotype and fluorochrome controls for PE and PE/Cy5.5. (**D**) Frequencies of γδ TCR^+^ and the WC1^+^ subset. (**E**) Expression of IL-10 and Foxp3 in the γδ TCR^+^ population. (**F**) WC1 phenotype of IL-10^+^ γδ TCR^+^ cells. (**G**) WC1.1 and (**H**) WC1.2 phenotype of IL-10^+^ γδ TCR^+^ WC1^+^ cells. Plots are representative of cells obtained from 16 different animals. Shaded histograms represent isotype and fluorochrome controls. (**I**) Frequency of CD4^+^, CD8^+^ and γδ TCR^+^ expressing IL-10. (**J**) Phenotype of γδ TCR^+^ IL-10^+^ cells based on WC1 expression. (**K**) Phenotype of γδ TCR^+^ IL-10^+^ WC1^+^ based on WC1-subgroup expression. Bars represent means of cells taken from animals (*n* = 16) and analyzed in duplicate. Error bars indicate SE of the means.

Despite γδ T cells representing a large component of the bovine lymphocyte system, the function of this cell population remains poorly understood. To explore a potential role in immune regulation, expression of Foxp3 and IL-10 by bovine PBMCs were analyzed ex vivo. Fig. 1A shows the typical frequency of CD4^+^ and CD8^+^ T cells in bovine peripheral blood. Of the CD4^+^ population, expression of IL-10 was not significantly higher compared with the isotype control staining, and <1% expressed Foxp3 as previously reported (40) (Fig. 1B). Fig. 1D shows the typical frequencies of γδ T cells and expression of WC1 in bovine peripheral blood. Between 7 and 15% (mean 11.43%) of all γδ TCR^+^ cells expressed IL-10, and the expression of Foxp3 was not significantly higher compared with the isotype control (Fig. 1E, 1I). Of the γδ T cells that express IL-10, 50% were WC1^−^ and 50% WC1^+^ (Fig. 1F, 1J), and of the WC1^+^ IL-10^+^ subset, most were WC1.2^+^ (Fig. 1G, 1H, 1K). The expression of CD25 was not significantly higher compared with the isotype control and did not correlate with expression of IL-10 (data not shown). Fig. 1C shows isotype and fluorochrome controls used to set the gates for intracellular staining.

**FIGURE 2.**
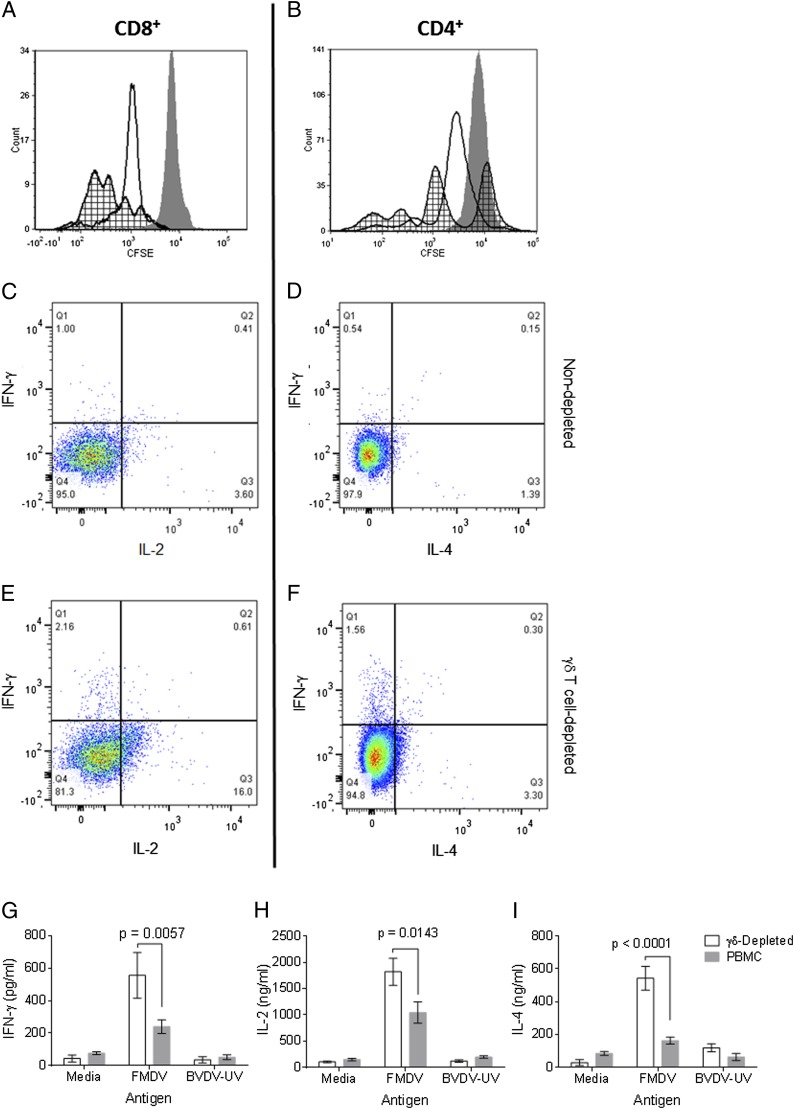
Depletion of γδ T cells increases Ag-specific responses in vitro. PBMCs from animals vaccinated with FMDV were depleted of the γδ TCR^+^ cells and cultured with FMDV Ag for 5 d. CD4^+^ and CD8^+^ FMDV-specific responses were analyzed by flow cytometry and compared with nondepleted samples. (**A**) Proliferation of CD8^+^ T cells and (**B**) proliferation of CD4^+^ T cells in response to FMDV measured by CFSE dilution. Gray histograms show nonspecific proliferation to media only; white histograms show Ag-specific proliferation in the presence of γδ T cells; hatched histograms show Ag-specific proliferation in γδ-depleted PBMCs. (**C**–**F**) IFN-γ, IL-2, and IL-4 expression in CD8^+^ (C and E) and CD4^+^ (D and F) T cells after stimulation with FMDV Ag in γδ T cell–depleted samples (E and F) or nondepleted samples (C and D). Histograms and dot plots are representative of cells from 10 different animals analyzed in duplicate. Only live/single events were analyzed. Quadrants were based on isotype staining (data not shown). Specific cytokine responses to FMDV and nonspecific responses to BVDV from culture supernatants were tested by ELISA and compared with nondepleted samples. (**G**) IFN-γ. (**H**) IL-2. (**I**) IL-4. Bars indicate means (*n* = 10, analyzed in duplicate), and error bars indicate SEMs.

**FIGURE 3.**
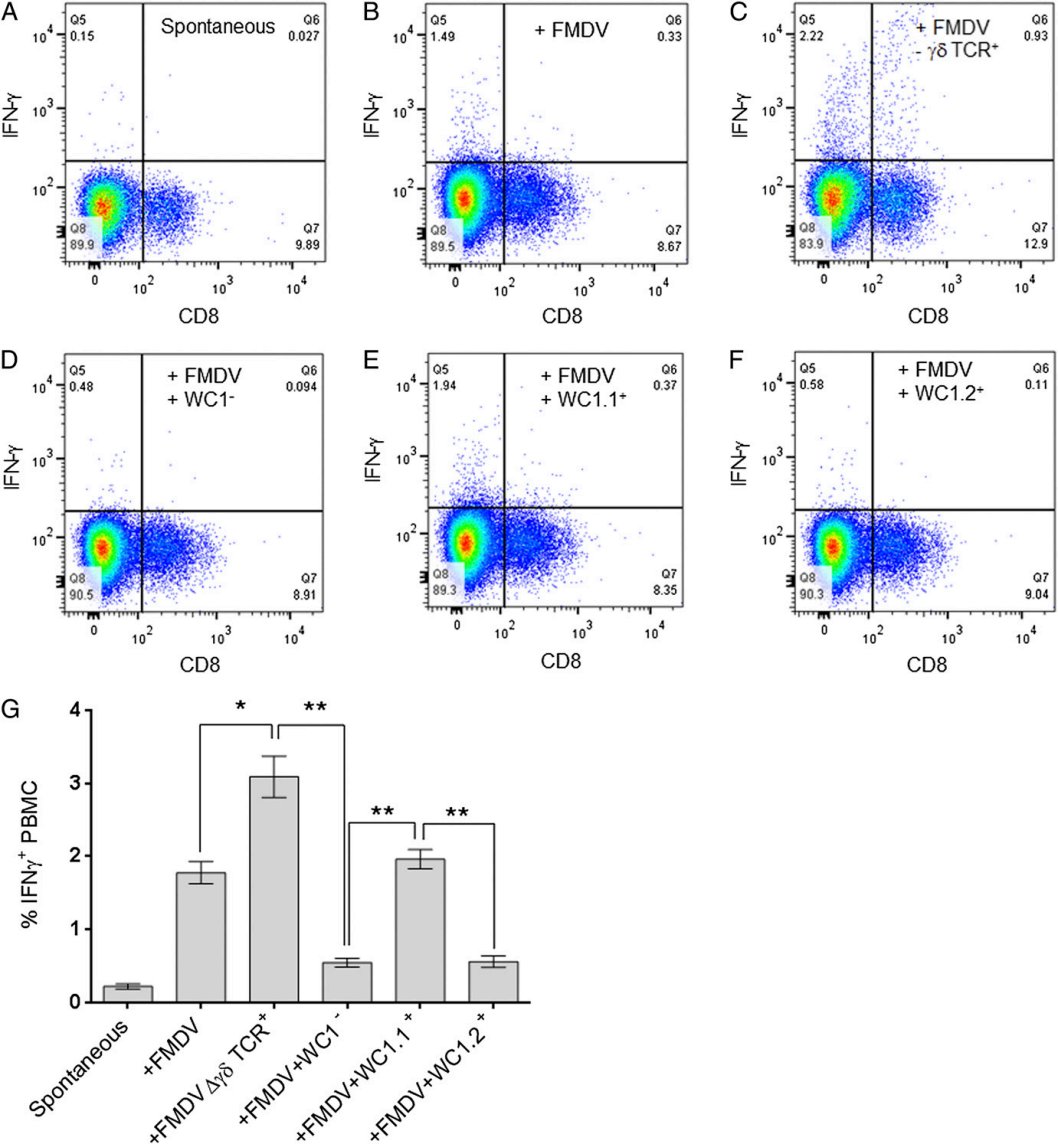
Inhibition of Ag-specific IFN-γ by γδ TCR^+^ subsets. PBMCs from FMDV-vaccinated animals were stimulated with media or FMDV Ag in the presence or absence of γδ TCR^+^ subsets. (**A**) IFN-γ expression to media only. (**B**) IFN-γ expression in PBMCs. (**C**) IFN-γ expression in γδ TCR-depleted PBMCs. (**D**) IFN-γ expression in γδ TCR-depleted PBMCs with WC1^−^ cells added back. (**E**) IFN-γ expression in γδ TCR-depleted PBMCs with WC1^+^ WC1.1^+^ WC1.2^−^ cells added back. (**F**) IFN-γ expression in γδ TCR-depleted PBMCs with WC1^+^ WC1.1^−^ WC1.2^+^ cells added back. MACS-purified γδ T cell subsets were added back at a ratio of 1 PBMC to 1 γδ T cell. Plots representative of cells taken from 10 animals and analyzed in duplicate. (**G**) Bar graph showing percentages of FMDV-specific IFN-γ^+^ cells in the presence of γδ T cell subsets. Bars represent means (*n* = 10); error bars represent SEMs; *p < 0.05, **p < 0.005.

To confirm that γδ T cells were involved in Ag-specific T cell regulation, we used cells from FMDV-vaccinated animals, because vaccination with inactivated virus has been shown to induce Ag-specific CD4^+^ and CD8^+^ T cells (16, 45). PBLs from FMDV-vaccinated cattle were depleted of γδ TCR^+^ cells, and FMDV-specific proliferation was measured in vitro. Fig. 2A, 2B show higher FMDV-specific proliferation of CD4^+^ and CD8^+^ T cells in samples depleted of γδ T cells. The depletion of γδ T cells had a statistically significant effect not only on proliferation, but also on the number of cells producing IFN-γ, IL-2, and IL-4. In the case of CD8^+^ T cells, depletion of γδ T cells resulted in a 5-fold increase in IL-2 and a 2-fold increase in IFN-γ expression (Fig. 2C, 2E). For CD4^+^ T cells, depletion of γδ T cells also resulted in increased IFN-γ and IL-4 expression (Fig. 2D, 2F). The quantity of cytokines released in these cultures was measured by ELISA, and there were statistically significant increases in the level of IFN-γ (*p* = 0.0057), IL-2 (*p* = 0.0143), and IL-4 (*p* < 0.001) in those samples that had been depleted of γδ T cells (Fig. 2G–I). To confirm that these effects were Ag specific, an irrelevant Ag (BVDV) was used in addition to media only. Responses to BVDV were similar to responses to media (Fig. 2G–I). To identify the γδ T cell subsets involved in this immune suppression, we depleted PBMCs from FMDV-vaccinated animals from all γδ T cells by MACS and stimulated them with FMDV Ag in the presence of autologous MACS-purified γδ T cell subsets described earlier using a ratio of 1 PBMC to 1 γδ T cell. Cultures containing WC1^−^ and WC1.2^+^ but not WC1.1^+^ γδ T cells showed decreased IFN-γ responses to FMDV (Fig. 3A–G). Although there was a reduction in the percentage of IFNγ^+^ cells in the +FMDV+WC1.1 group compared with the γδ-depleted group, this was not statistically significant (*p* = 0.1713). These results indicate that bovine γδ T cells have potent inhibitory functions.

### Expansion of IL-10 – expressing γδ T cells in vitro

**FIGURE 4.**
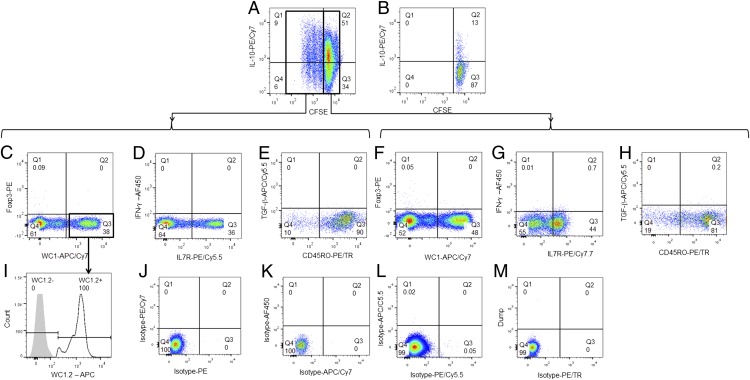
Expansion of IL-10–expressing γδ T cells in vitro. γδ T cells were MACS sorted and tested for their ability to expand in vitro. (**A**) γδ T cells cultured with autologous monocytes (CD14^+^) and media only for 5 d. (**B**) γδ T cells cultured with rIL-2, anti-CD3, and anti-CD28 for 5 d. Phenotype of CFSE^low^ (**C**–**E**) and CFSE^high^ (**F**–**H**) γδ T cells: (C and F) expression of Foxp3/WC1; (D and G) IFN-γ/IL-7R; (E and H) TGF-β/CD45RO; and (**I**) WC1-subgroup phenotype of CFSE^low^ γδ TCR^+^ cells. Isotype controls (**J**–**M**) for each of the fluorochromes used is also shown. Plots are representative of cells from 10 different animals analyzed in duplicate. Only live/singe events were analyzed.

For expansion in vitro, human and murine Tregs (CD4^+^ CD25^+^ Foxp3^+^) require IL-2, engagement of the TCR via cross-linking with anti-CD3, and induction of Foxp3 by TGF-β (46, 47). To identify whether bovine IL-10–expressing γδ T cells could be expanded the same way, we cultured purified γδ TCR^+^ cells with autologous peripheral blood monocytes (CD3^−^sIg^−^MHCII^+^CD14^+^) alone or with rIL-2, anti-CD3, and anti-CD28 as described previously (34). Between 7 and 15% of γδ T cells cultured with monocytes and media only proliferated (Fig. 4A) and ∼50% of CFSE^low^ γδ TCR^+^ cells were IL-10^+^; cells cultured with rIL-2, anti-CD3, and anti-CD28 did not proliferate, and most cells died within 36 h of culture (Fig. 4B). Positively sorted γδ T cells cultured in the absence of monocytes did not survive >24 h in culture (data not shown). Both CFSE^low^ and CFSE^high^ γδ T cells were WC1^−^ and WC1^+^ and Foxp3^−^ (Fig. 4C, 4F); within the WC1^+^ cells, only the WC1.2^+^ subset proliferated and expressed IL-10 (Fig. 4I), mirroring the results obtained ex vivo.

Under the conditions tested, both proliferating and nonproliferating IL-10^+^ γδ TCR^+^ cells were found to be IFN-γ^−^, either IL7R^−^ or IL7R^+^, TGF-β^−^, and most were CD45RO^+^ (Fig. 4D–H). To confirm that selection by MACS did not have a nonspecific effect on the expansion of IL-10–producing γδ T cells, we negatively selected peripheral blood γδ TCR^+^ cells by MACS or flow sorted and cultured them with autologous monocytes as described earlier with similar results (data not shown).

To define the monocyte-derived signals required for the expansion of IL-10^+^ γδ T cells, we cultured MACS-sorted γδ T cells with gamma-irradiated autologous monocytes and in the presence of blocking Abs to various cytokines, individually and in combination. Consistent with previous observations, γδ T cells proliferated in culture with autologous monocytes but did not survive in the absence of monocytes, and <10% survival was observed in the presence of autologous B cells (Table I). Anti–IL-4 or TNF-α blocking Abs did not have a significant effect on expression of IL-10 or proliferation of γδ cells. Anti–TGF-β and anti–IL-10 individually and in combination reduced proliferation and expression of IL-10 in γδ T cells, and anti–IL-10 had the most significant effect (Table I). These effects are partly explained by the increase in γδ T cell death after incubation with anti–IL-10 and anti–TGF-β. Significant increases in IFN-γ were only observed when γδ T cells were stimulated with mitogen. To investigate whether contact with monocytes was required to expand IL-10–producing γδ T cells, we used a Transwell system where irradiated CD14^+^ were placed in the lower chamber and autologous γδ T cells in the upper chamber. By preventing the physical contact between γδ T cells and monocytes, expansion of IL-10^+^ γδ T cells did not occur and most cells died within the first 2 days of the assay (Table I). These results show that in vitro expansion of bovine IL-10^+^ γδ T cells requires IL-10, TGF-β, and contact with APCs, in this case, peripheral blood CD14^+^ monocytes.

**Table I.** Cytokine stimulation required for the expansion of IL-10–expressing γδ T cells in vitro

Blocking Ab/Stimulus	Monocytes	% Live/CFSE^low^ Cells	% Live/IL-10^+^	% Live/IFN-γ^+^	% Dead Cells
Media only	Y	16 ± 4	62 ± 12	2 ± 2	13 ± 5
Media only	N	0*	1.1 ± 2*	0	95 ± 5*
rbo IL-10 (10 U/ml)	Y	27 ± 10	50 ± 8	2 ± 2	12 ± 6
Anti–IL-4	Y	16 ± 10	51 ± 9	1 ± 2	14 ± 4
Anti–TGF-β	Y	7 ± 3*	12 ± 2*	3 ± 2	13 ± 6
Anti–TNF-α	Y	17 ± 5	51 ± 7	1 ± 2	7 ± 2
Anti–IL-10	Y	2 ± 2*	3 ± 2*	3 ± 2	23 ± 12*
Anti–IL-4 + anti–TGF-β	Y	11 ± 4	12 ± 2*	0.9 ± 1	13 ± 6
Anti–IL-4 + anti–TNF-α	Y	18 ± 8	49 ± 9	1 ± 2	7 ± 6
Anti–IL-4 + anti–IL-10	Y	5 ± 8*	2 ± 2*	3 ± 2	21 ± 7
Anti–TGF-β + anti–TNF-α	Y	11 ± 5	13 ± 4*	2.±2	23 ± 2*
Anti–TGF-β + anti–IL-10	Y	1 ± 2*	1 ± 2*	4 ± 2	95 ± 4*
Anti–TNF-α + anti–IL-10	Y	2 ± 3*	3 ± 2*	1 ± 2	83 ± 8*
Anti–IL-4 + α-TGF-β + α-TNF-α	Y	15 ± 7	13 ± 5*	2 ± 2	15 ± 7
Anti–TGF-β + anti–TNF-α + anti–IL-10	Y	2 ± 3*	0.5 ± 2*	3 ± 3	63 ± 9*
Anti–IL-4 + anti–TGF-β + anti–TNF-α + anti–IL-10	Y	2 ± 2*	0.8 ± 2*	3 ± 3	63 ± 8*
Anti–TGF-β + anti–TNF-α + anti–IL-10	N	1 ± 2*	1 ± 2*	2 ± 2	58 ± 8*
Media only + B cells	N	7 ± 3*	8 ± 3*	1 ± 2	68 ± 5*
Media only + T cells	N	1 ± 1*	0.5 ± 2*	2 ± 4	88 ± 10*
PMA/Ion	Y	31 ± 5	11 ± 5*	12 ± 5*	28 ± 11
Media only: Transwell	Y	3.1 ± 2*	1 ± 2*	0	88 ± 12
Anti–IL-10: Transwell	Y	1.1 ± 2*	1 ± 1*	0	92 ± 9

Purified γδ T cells were cocultured with irradiated autologous monocytes (CD14^+^), B cells, or T cells for 5 d, in the presence or absence of recombinant cytokines or blocking Abs. Proliferation was measured by CFSE dilution; intracellular cytokines and viability were analyzed by flow cytometry. Data show means of cells obtained from 10 different animals and analyzed in duplicate ± error of the means.

*p < 0.05, statistically significant difference compared with media only + monocytes.

PMA, phorbol 12-myristate 13-acetate.

In light of these results, and for all subsequent experiments, positively sorted γδ T cells were expanded in vitro for 5 d by coculture with autologous monocytes and without any additional cytokines.

### In vitro–expanded γδ T cells modulate proliferation and function of CD4^+^ and CD8^+^ T cells after specific and nonspecific stimulation

**FIGURE 5.**
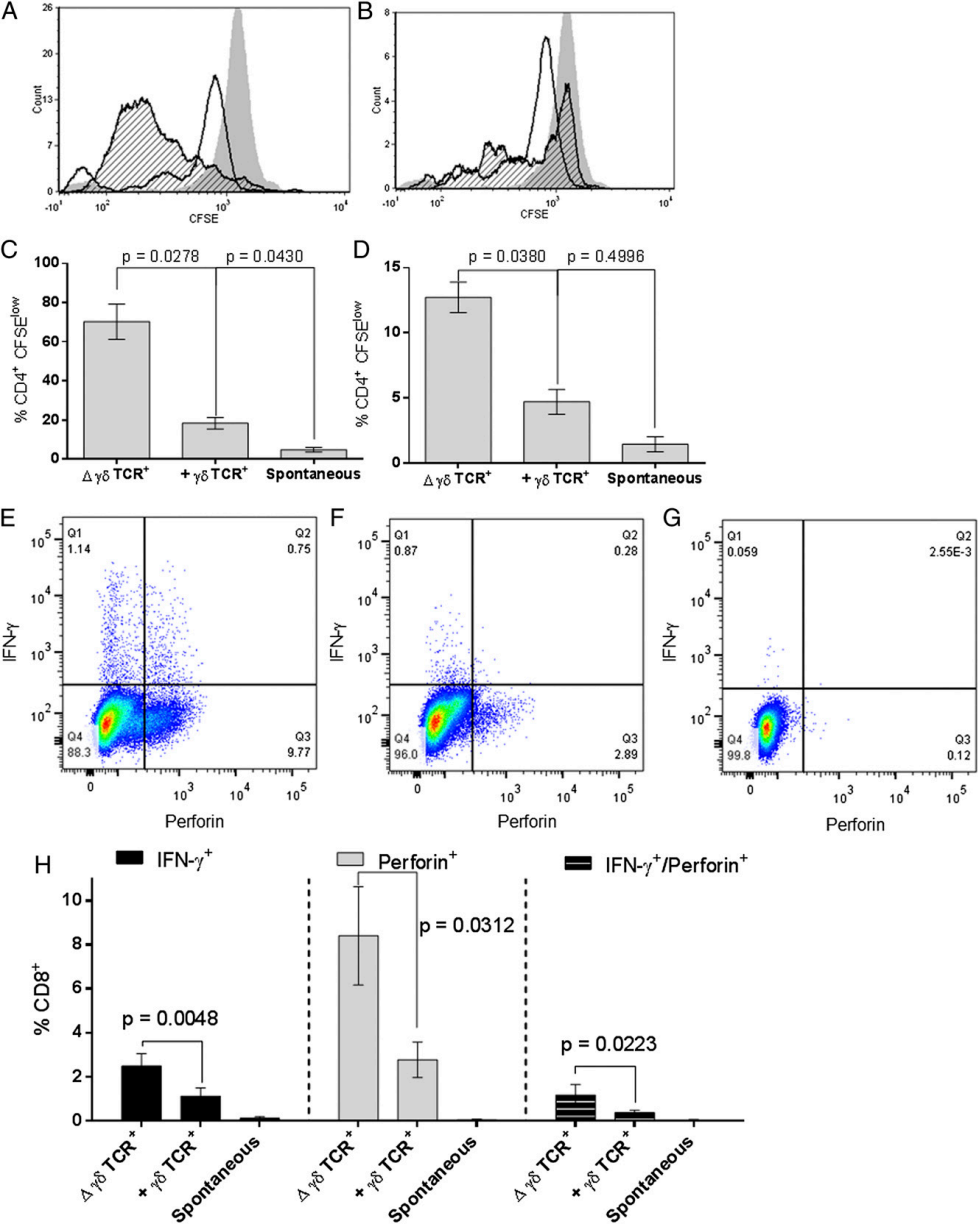
In vitro–expanded γδ T cells inhibit nonspecific and Ag-specific proliferation and function of αβ T cells. (**A**) CD4^+^ T cells were cultured with autologous CD14^+^ cells in the presence of IL-2, anti-CD3, and anti-CD28 in the absence (gray histogram shows spontaneous proliferation without stimulus. hatched histogram) or presence (white histogram) of in vitro–expanded γδ T cells. (**B**) CD4^+^ T cells were cultured with autologous CD14^+^ cells loaded with FMDV Ag and cultured in the absence (hatched histogram) or presence (white histogram) of in vitro–expanded γδ T cells. Gray histogram shows spontaneous proliferation without stimulus. Histograms are representative samples of cells from six different animals analyzed in duplicate. (**C** and **D**) Bar graphs showing nonspecific (IL-2, anti-CD3, anti-CD28–driven) (C) and FMDV Ag-specific (D) proliferation of CD4^+^ T cells in the presence or absence of in vitro–expanded γδ T cells. Spontaneous proliferation to culture media is also shown. Bars indicate means (*n* = 6), and error bars indicate SDs. (**E**–**G**) CD8^+^ T cells cultured with autologous CD14^+^ cells loaded with FMDV Ag and cultured in the absence (E) or presence (F) of in vitro–expanded γδ T cells were stained for intracellular IFN-γ and perforin, and analyzed by flow cytometry. (G) Isotype and fluorochrome controls of intracellular staining are shown. Data are representative of cells obtained from six different animals and analyzed in duplicate. (**H**) Bar graph showing a reduction in cytokine expression in FMDV-specific CD8^+^ T cells in the presence of in vitro–expanded γδ T cells. Spontaneous cytokine expression to culture media is also shown. Bars indicate means (*n* = 6), and error bars indicate SEMs.

To investigate whether in vitro–expanded IL-10–expressing γδ T cells were capable of regulating proliferation and function of T cells, we cultured purified CD4^+^ T cells with an equal number of autologous CD14^+^ monocytes in the presence of rIL-2, anti-CD3, and anti-CD28. After the addition of in vitro–expanded, autologous γδ T cells at a ratio of 10:1 CD4^+^ T cells, polyclonal proliferation of CD4^+^ T cells was reduced (*p* = 0.0278; Fig. 5A, 5C) compared with CD4^+^ T cells expanded in the absence of γδ T cells. To confirm this effect in Ag-specific T cell proliferation, we cultured purified CD4^+^ T cells from FMDV-vaccinated animals with autologous CD14^+^ cells loaded with FMDV Ag. In the absence of γδ T cells, CD4^+^ T cells proliferated in response to FMDV Ag. However, after the addition of in vitro–expanded autologous γδ T cells, FMDV-specific CD4^+^ proliferation was reduced (*p* = 0.0380; Fig. 5B, 5D).

The effect of in vitro–expanded γδ T cells on CTL phenotype was also investigated. Purified CD8^+^ T cells were cultured with an equal number of autologous CD14^+^ cells that had been loaded with FMDV Ag, in the presence or absence of in vitro–expanded autologous γδ T cells. There was a reduction in IFN-γ^+^ (*p* = 0.0048), perforin-positive (*p* = 0.0312), and double perforin/IFN-γ^+^ (*p* = 0.0223) FMDV-specific CD8^+^ T cell frequencies in the presence of in vitro–expanded γδ T cells (Fig. 5E–G). These results confirm the biological function of in vitro–expanded γδ T cells, reducing Ag-specific and nonspecific proliferation and function of CD4^+^ and CD8^+^ T cells.

### Suppressive γδ T cells are induced by APCs of various phenotypes

**FIGURE 6.**
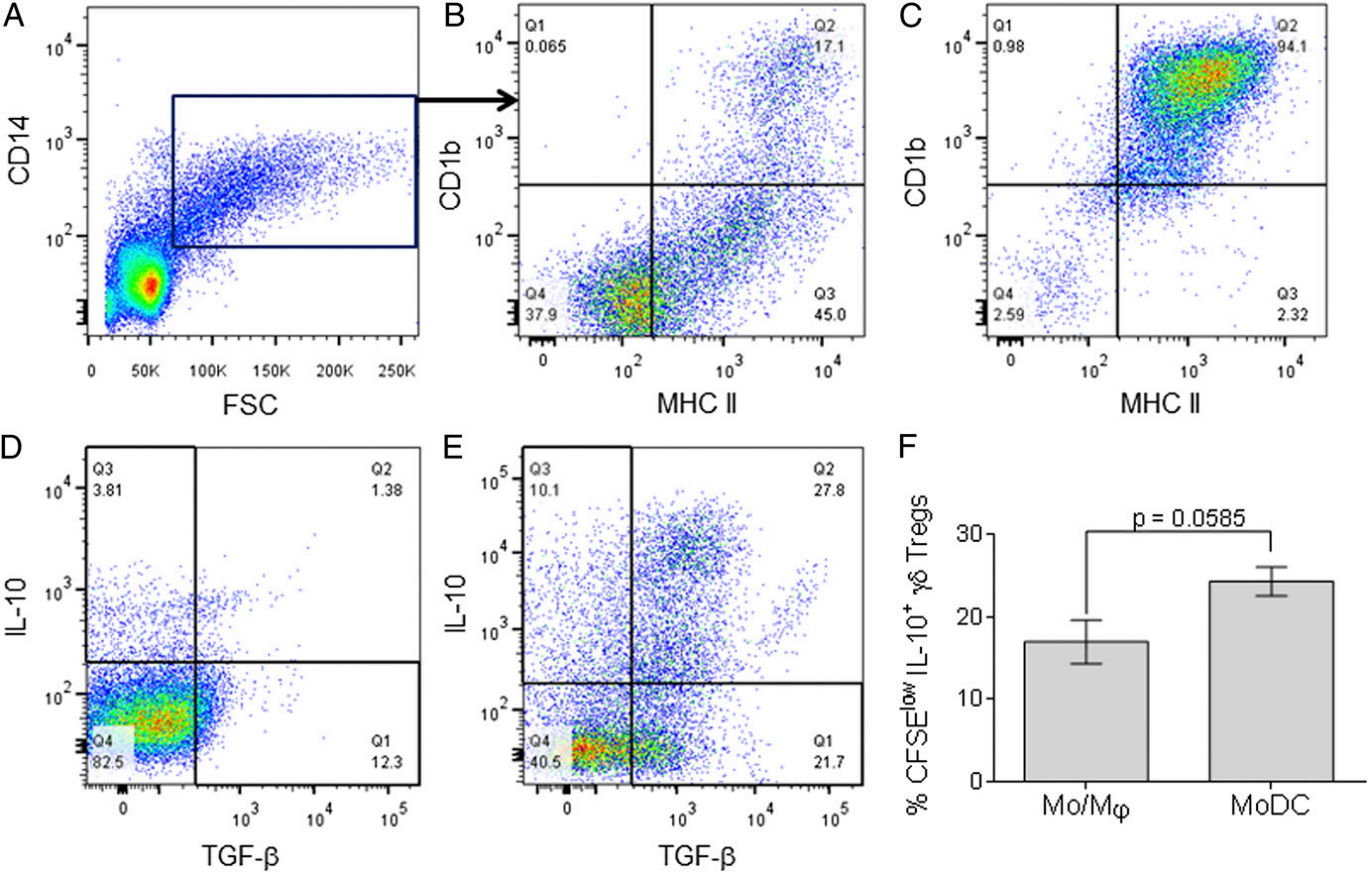
Monocyte/macrophages and MoDCs express IL-10 and TGF-β, and induce proliferation of IL-10^+^ γδ T cells. Peripheral blood monocyte/macrophages expressing CD14 (**A**) and expression of MHC class II/CD1b (**B**) in CD14^+^ cells. After a 3-d culture in the presence of GM-CSF and IL-4, the cells increased expression of MHC class II and CD1b (**C**). IL-10 and TGF-β expression in ex vivo CD14^+^ cells (**D**) and cultured MoDCs (**E**). Both monocyte/macrophages and MoDCs induced the expansion of autologous IL-10–expressing γδ T cells (**F**). Bar graph shows means (*n* = 10) and error bars indicate SEMs. Representative plots of cells obtained from 10 different animals analyzed in duplicate. Quadrants were placed based on isotype and fluorochrome controls.

Efficient mechanisms for the induction of tolerance or maintenance of homeostasis are necessary at sites of infection or vaccination, where APCs process and present both self- and non–self-Ags. In vitro–matured MoDCs have been used as a model to study DC biology and have been shown to be required for the expansion of Tregs in mice and humans. Bovine peripheral blood CD14^+^ cells (Fig. 6A) expressed low to high levels of MHC class II, and most were CD1b^−^ (Fig. 6B). Less than 5% of these cells expressed IL-10, ∼13% expressed TGF-β, and <2% were double positive (Fig. 6D). After maturation with rIL-4 and GM-CSF, the phenotype of CD14^+^ cells changed with the majority expressing the phenotype MHC^high^ CD1b^+^ (Fig. 6C), and these were termed MoDCs. More than 10% of these cells expressed IL-10, 20% expressed TGF-β, and up to 30% of MoDCs were IL-10^+^TGF-β^+^ (Fig. 6E). The ability of CD14^+^ monocyte/macrophages and MoDCs to expand IL-10^+^ γδ T cells was assessed in vitro. In the absence of any additional stimulus, there was a greater expansion of IL-10^+^ γδ T cells when cultured with autologous MoDCs than with monocyte/macrophages, but this difference was not statistically significant (*p* = 0.0585; Fig. 6F). About 15% of CD14^+^ cells expressed IL-10 ex vivo (Fig. 6D), and this remains the same when cultured for 3 d in media only (Supplemental Fig. 1). However, when cocultured with γδ T cells (and in the absence of GM-CSF and IL-4), this percentage increased to 40–50% (Supplemental Fig. 1) and may be explained by the fact that IL-10 secreted by γδ T cells induce a positive-feedback mechanism on the secretion of IL-10 by the APCs in the coculture assay.

**FIGURE 7.**
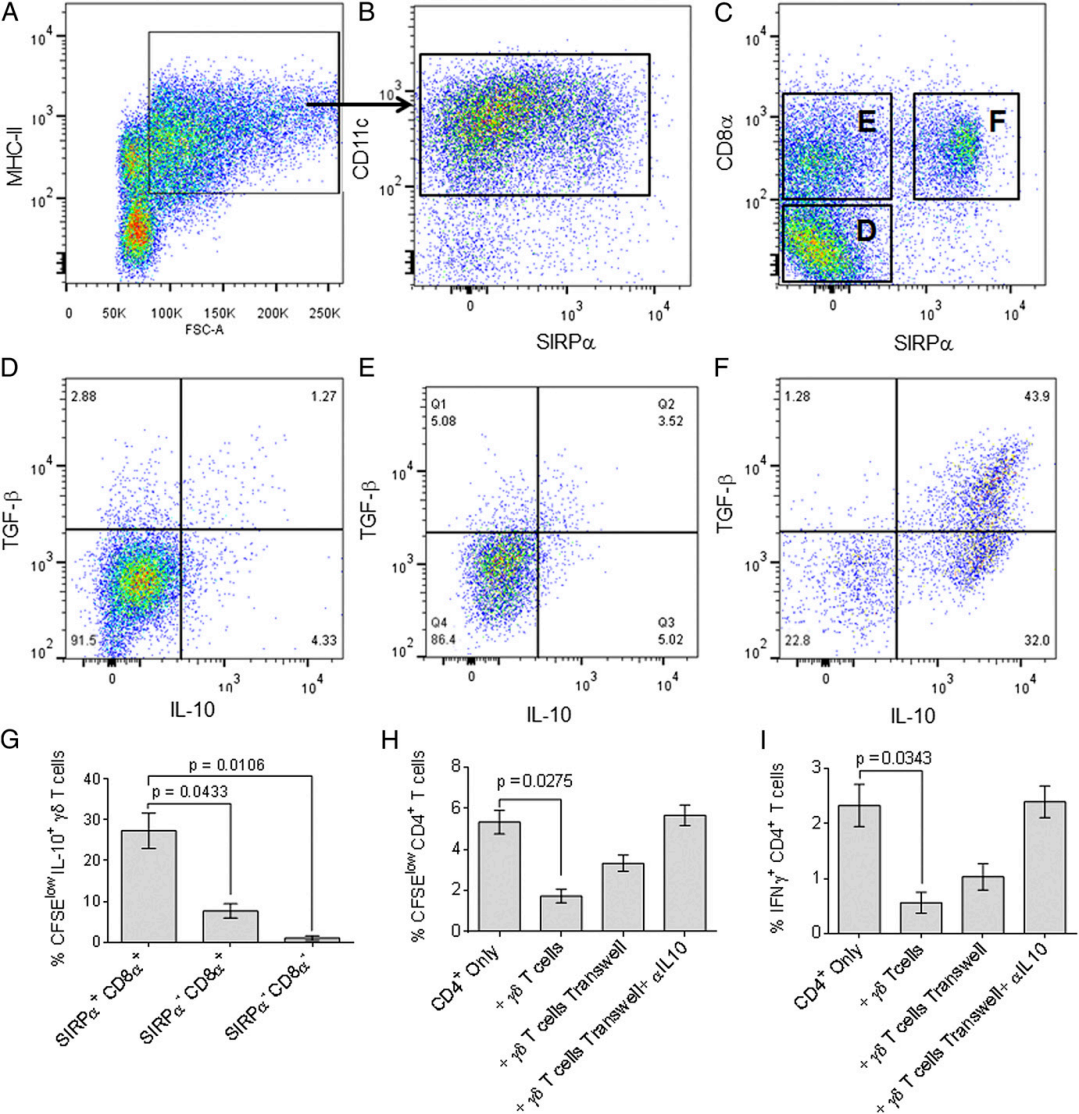
CD8α^+^ SIRPα^+^ lung-resident DCs express cytokines that induce γδ T cells with suppressive phenotype. Phenotypic analysis of bovine lung DC subsets includes FSC^high^ MHC class II (**A**), SIRPα and CD11c (**B**), and SIRPα and CD8α (**C**). Cells were fixed, permeabilized, and stained for intracellular TGF-β and IL-10. DCs were gated on SIRPα^−^ CD8α^−^ double negatives (**D**), SIRPα^−^ CD8α^+^ single positives (**E**), and SIRPα^+^ CD8α^+^ double positive (**F**). Dot plots are representative of tissues from six different animals. (**G**–**I**) SIRPα^+^ CD8α^+^ lung DCs are capable of inducing γδ T cells with suppressive phenotype. (G) Subpopulations of lung-resident DCs were FACS sorted and cocultured with autologous MACS-sorted γδ TCR^+^ T cells for 5 d. Proliferation was analyzed by CFSE dilution, and cells were stained for intracellular IL-10. (H) FMDV-specific proliferation of CD4^+^ T cells in the absence or presence of in vitro–expanded γδ T cells by SIRPα^+^ CD8^+^ lung DCs and in Transwell plates with or without blocking anti–IL-10. (I) FMDV-specific IFN-γ responses in CD4^+^ T cells in the presence or absence of in vitro–expanded autologous γδ T cells SIRPα^+^ CD8^+^ lung DCs and in Transwell plates with or without blocking anti–IL-10. Bars indicate means of cells from four different animals analyzed in triplicate, and error bars indicate SEMs.

The regulation of T cell responses in mucosal surfaces, which are constantly stimulated by environmental Ags, is important because there must be a tight control of T cell proliferation in response to nonpathogenic stimuli, autoantigens, and superantigens. To understand how homeostasis is maintained in the bovine lung, we identified lung-resident DC subsets responsible for secreting activation signals that are required for regulatory γδ T cell proliferation and function. Bovine lung DCs (FSC^high^ MHCII^+^, CD11c^+^; Fig. 7A, 7B) could be subdivided into SIRPα^+^ or SIRPα^−^ DCs (Fig. 7B). In turn, these two populations could be subdivided further on the expression of CD8α, and three populations were evident: SIRPα^−^ CD8α^−^, SIRPα^−^ CD8α^+^, and SIRPα^+^ CD8α^+^ (Fig. 7C). In the SIRPα^−^ CD8α^−^ population, <5% expressed IL-10 and <5% was double positive for TGF-β and IL-10 (Fig. 7D). This was also the case for the SIRPα^−^ CD8α^+^ population (Fig. 7E). In contrast, ∼80% of the SIRPα^+^ CD8α^+^ cells expressed IL-10, >40% produced TGF-β, and ∼40% were double positive for expression of TGF-β and IL-10 (Fig. 7F).

The various populations of lung DCs—SIRPα^+^ CD8α^+^ double positives, SIRPα^−^ CD8α^+^ single positives, and SIRPα^−^ CD8α^−^ double negatives—were purified and cultured with autologous peripheral blood γδ TCR^+^ cells in the absence of any other stimulus, to test the ability of these different lung DC subsets to induce proliferation of IL-10–expressing γδ T cells. Only those γδ T cells cocultured with CD8α^+^ SIRPα^+^ DCs were able to proliferate and express IL-10 (Fig. 7G). To confirm that these expanded γδ T cells had an inhibitory phenotype, they were cultured together with CFSE-labeled CD4^+^ T cells from FMDV-vaccinated animals and autologous MoDCs, which had been loaded with FMDV Ag. FMDV-specific CD4^+^ T cell proliferation and the frequency of FMDV-specific IFN-γ^+^ CD4^+^ T cells were reduced (*p* = 0.0275 and *p* = 0.0343, respectively) in the presence of γδ T cells that had been expanded by culture with SIRPα^+^CD8^+^ lung DCs, compared with CD4^+^ cells cultured in the absence of expanded γδ T cells (Fig. 7H, 7I). To investigate whether the observed effects were mediated by contact with the in vitro–expanded γδ T cells or were due to soluble factors, we placed irradiated CD14^+^ monocytes and γδ T cells in the lower chamber and CD4^+^ T cells in the upper chamber of Transwell plates. The frequency of FMDV-specific proliferation and IFN-γ^+^ CD4^+^ T cells was lower, but not statistically significant, in those samples containing γδ T cells separated by the Transwell plate compared with those samples without γδ T cells (Fig. 7H, 7I). Immune suppression was completely reversed by the addition of blocking anti–IL-10 Abs to the Transwell plates, with IFN-γ responses and proliferation similar to those obtained in the absence γδ T cells (Fig. 7H, 7I). These results indicate that direct contact as well as IL-10 production by γδ T cells is required for suppression of Ag-specific responses.

**FIGURE 8.**
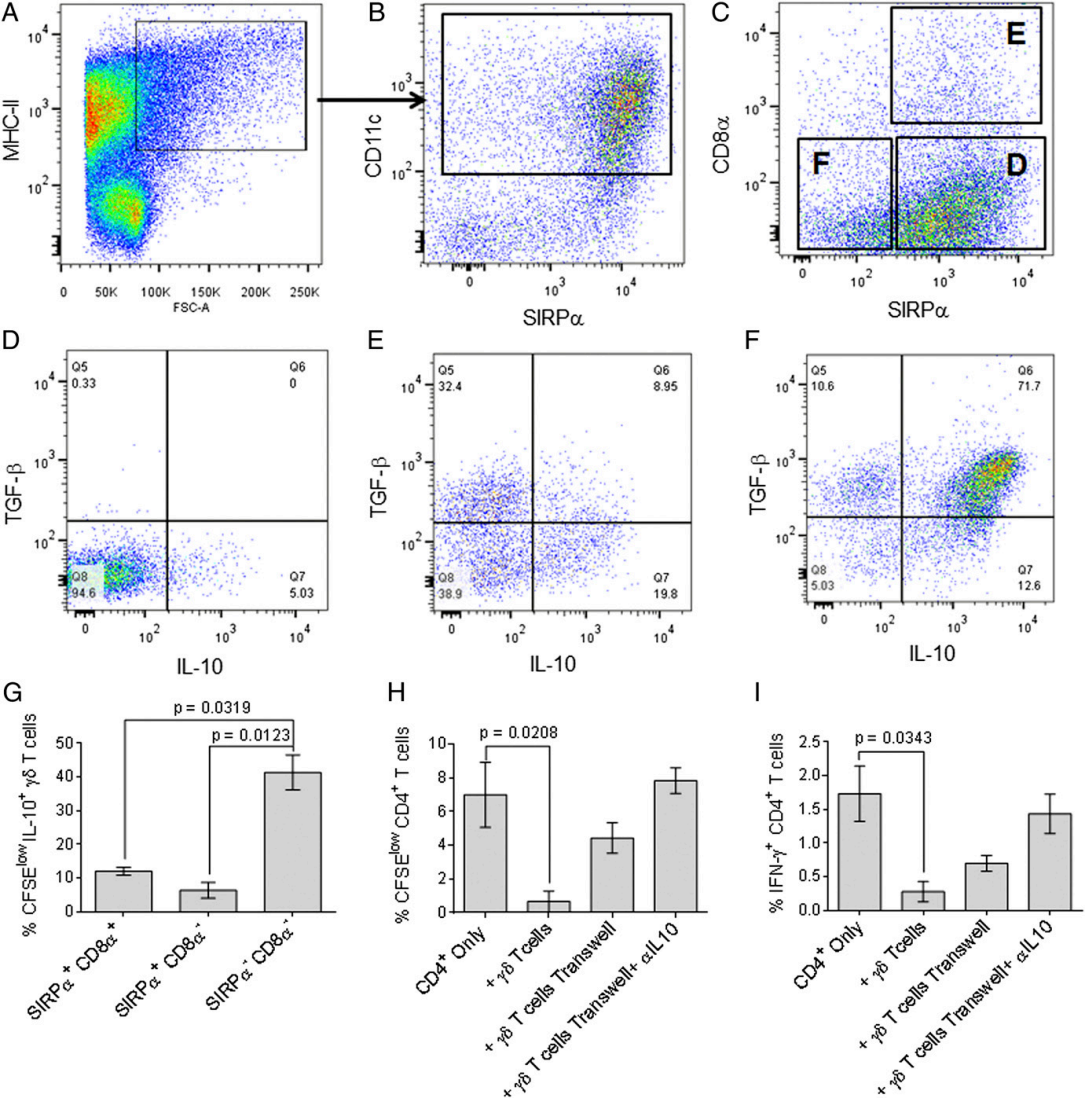
CD8α^−^ SIRPα^−^ ALDCs express cytokines that induce γδ T with a regulatory phenotype. Phenotypic analysis of bovine ALDC subsets includes FSC^high^ MHC class II (**A**), SIRPα and CD11c (**B**), and SIRPα and CD8α (**C**). Cells were fixed, permeabilized, and stained for intracellular TGF-β and IL-10. DCs were gated on SIRPα^+^ CD8α^−^ (**D**), SIRPα^+^ CD8α^+^ (**E**), and SIRPα^−^ CD8α^−^ double negative (**F**). Dot plots are representative of cells from five different animals. (**G**–**I**) SIRPα^−^ CD8α^−^ ALDCs are capable of inducing γδ T cells with suppressive phenotype. (G) Subpopulations of ALDCs were FACS sorted and cocultured with autologous MACS-sorted γδ TCR^+^ T cells for 5 d. Proliferation was measured by CFSE dilution and intracellular staining of IL-10 by flow cytometry. (H) FMDV-specific proliferation of CD4^+^ T cells in the absence or presence of in vitro–expanded autologous γδ T cells by SIRPα^−^ CD8α^−^ ALDCs and in Transwell plates with or without blocking anti–IL-10. (I) FMDV-specific IFN-γ responses in CD4^+^ T cells in the presence or absence of in vitro–expanded autologous γδ T cells SIRPα^−^ CD8α^−^ ALDCs and in Transwell plates with or without blocking anti–IL-10. Bars indicate means of cells from four different animals analyzed in triplicate, and error bars indicate SEMs.

Vaccination through the skin is the most commonly used route to deliver vaccine Ags. We therefore looked at various ALDC subsets, collected by cannulating lymphatic vessels that drain the skin of cattle (28) for their capacity to induce IL-10^+^ γδ T cells. As described earlier, ALDCs were defined as FSC^high^ MHCII^high^ CD11c^+^ DEC205^+^ (15) (Fig. 8A). Two subpopulations were then identified based on surface expression of SIRPα with ∼70% of ALDCs being SIRPα^+^, and the remainder being SIRPα^low^ and SIRPα^−^ (28) (Fig. 8B). In contrast with lung DCs, expression of CD8α was very low: <10% of the SIRPα^+^ population also expressed CD8α, and the SIRPα^−^ population was also CD8α^−^ (Fig. 8C). The expression of TGF-β and IL-10 in these ALDC subsets was analyzed: within the SIRPα^+^ CD8α^−^ population, <5% was IL-10^+^ (Fig. 8D). The SIRPα^+^ CD8α^+^ population of ALDCs contained distinct cytokine-expressing subsets: ∼40% was double negative for TGF-β and IL-10, 20% was IL-10^+^, 30% was TGF-β^+^, and <10% was double positive (Fig. 8E). Of the SIRPα^−^ CD8α^−^ population, ∼5% was TGF-β and IL-10 double negative, 10% was single positive, and >70% was double positive (Fig. 8F). The ability of these different ALDC subsets to induce proliferation of IL-10^+^ γδ T cells was measured in vitro. The populations of ALDCs—SIRPα^+^ CD8α^+^ double positives, SIRPα^+^ CD8α^−^ single positives, and SIRPα^−^ CD8α^−^ double negatives—were flow sorted and cultured with autologous γδ TCR^+^ cells in the absence of any other stimulus. γδ T cells cocultured in the presence of SIRPα^−^ CD8α^−^ ALDCs showed increased proliferation compared with those cocultured with SIRPα^+^ CD8α^+^ double-positive ALDCs (*p* = 0.0319) or those cultured with SIRPα^+^ CD8α^−^ (*p* = 0.0123; Fig. 8G). To confirm that these expanded γδ T cells had an inhibitory phenotype, we cultured CFSE-labeled CD4^+^ T cells from FMDV-vaccinated animals with autologous MoDCs that had been loaded with FMDV Ag, and the in vitro–expanded γδ T cells were added to these cultures. CD4^+^ T cells that were cultured in the presence of γδ T cells showed less proliferation (*p* = 0.0208) and a reduced IFN-γ response (*p* = 0.0343) compared with those cells cultured in the absence of γδ T cells (Fig. 8H, 8I). To investigate whether the observed effects were contact specific or due to soluble factors, we placed γδ T cells in the lower chamber and CD4^+^ T cells in the upper chamber of Transwell plates as described earlier. The frequency of FMDV-specific proliferation and IFN-γ^+^ CD4^+^ T cells was lower, but not statistically significant, in those samples containing γδ T cells separated by the Transwell plate compared with those samples without γδ T cells (Fig. 8H, 8I). Immune suppression by the γδ T cells was completely reversed by the addition of blocking anti–IL-10 Abs to the Transwell plates (Fig. 8H, 8I). These results indicate that direct contact as well as IL-10 production by γδ T cells is required for suppression of Ag-specific responses, and the ability to induce proliferation of γδ T cells with a regulatory phenotype depends on the origin and phenotype of the APC.

### Recombinant MVA induces the generation of IL-10–expressing γδ T cells in vitro

**FIGURE 9.**
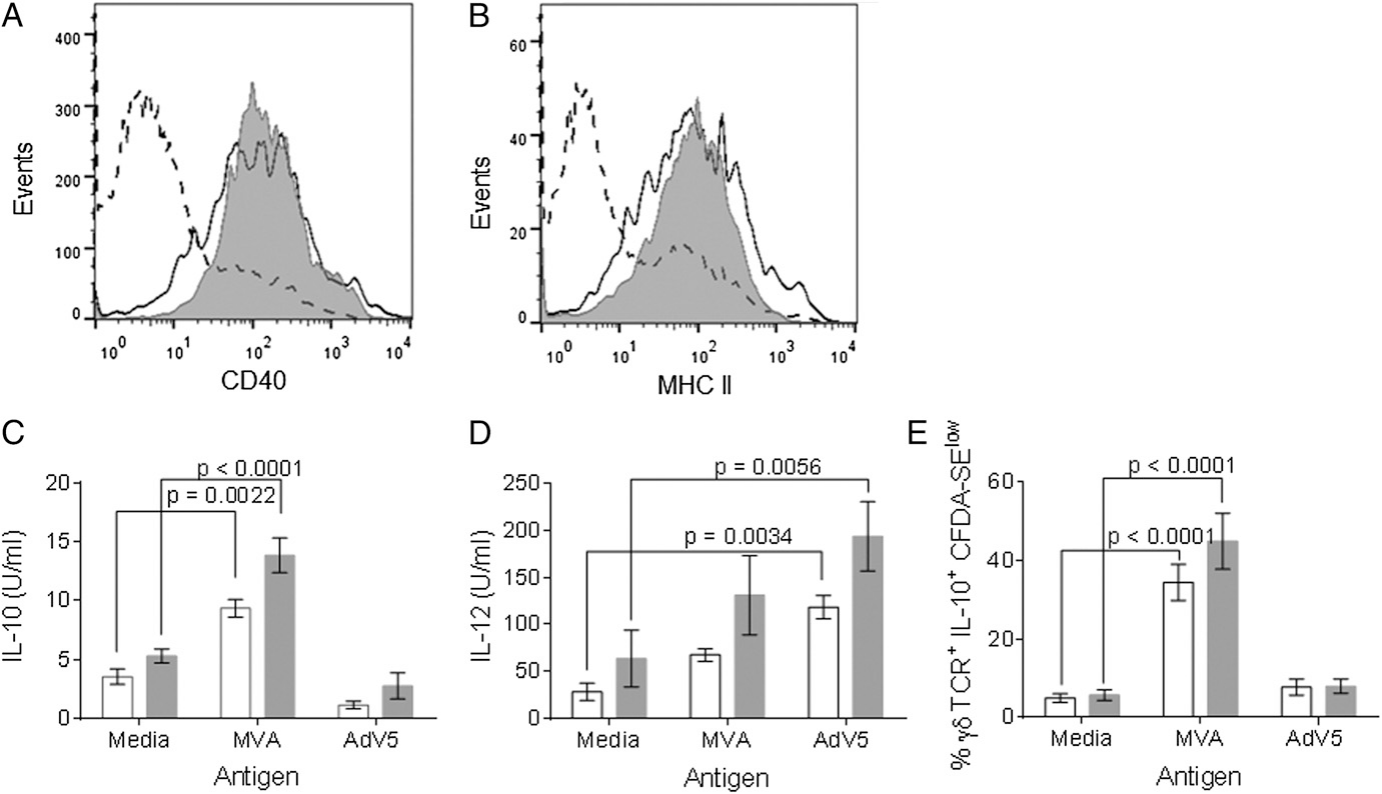
MVA induces the production of IL-10–expressing γδ T cells. FACS-sorted ALDCs were infected with MVA-GFP (dotted histograms) transduced with AdV5-GFP (white histograms) or mock infected (gray histograms). (**A**) Surface expression of CD40 and (**B**) MHC class II. Histograms are representative samples of cells from six different animals. Culture supernatants from monocytes (white bars) or ALDCs (gray bars) were analyzed by ELISA for the presence of (**C**) IL-10 and (**D**) IL-12. (**E**) FACS-sorted ALDCs (from A and B) were cultured with autologous CFDA-SE–labeled PBMCs (ratio of 10 PBMC:1 γδ T cell), and lymphocyte proliferation, T cell subsets, and intracellular cytokines were measured by flow cytometry. Bars indicate means of cells obtained from six different animals and tested in duplicate, and error bars indicate SEMs.

MVA and human replication-deficient AdV5 are two of the most commonly used viral vector systems being evaluated for therapeutic and prophylactic vaccination in both humans and livestock. We have previously reported that ALDC infected with MVA, but not AdV5, reduces cell-surface expression of MHC class II, CD40, and CD86 (15) and low expression levels of these molecules on APCs have been associated with the generation and expansion of regulatory cells in other systems (48). Therefore, we tested the hypothesis that MVA-infected DCs would induce the expansion of IL-10–expressing γδ T cells. Purified CD14^+^ cells or ALDCs (FSC^high^ DEC205^+^ MHCII^+^, CD11c^+^) were infected with either MVA-GFP (multiplicity of infection = 1) or AdV5-GFP (multiplicity of infection = 100) and after an overnight culture, the expression of CD40 and MHC class II was reduced in those cells infected with MVA-GFP (Fig. 9A, 9B and data not shown). By contrast, exposure to AdV5 had no effect on the expression of these molecules. We then measured the amount of IL-10 and IL-12 present in the culture supernatants. There was a significant increase (*p* < 0.0001) in the amount of IL-10 produced by ALDCs and CD14^+^ cells in response to infection with MVA (Fig. 9C). Conversely, there was a significant increase (*p* < 0.0001) in the amount of IL-12 produced by both APC types in response to AdV5, but not to MVA when compared with uninfected cells (Fig. 9D). To assess the effect of vaccine vectors on IL-10–expressing γδ T cells, we cultured MACS-sorted monocytes or FACS-sorted ALDCs with AdV5 or MVA and with autologous CFSE-labeled PBMCs. Fig. 9E shows that after a 5-d culture, the number of proliferating IL-10^+^ γδ T cells was higher in those samples that had been infected with MVA (*p* < 0.0001 compared with culture media and AdV5).

## Discussion

γδ T cells represent a minor percentage of the peripheral lymphocyte pool in humans and rodents. In contrast, they represent a major lymphocyte subset in cattle and can constitute up to 60% of the circulating T cells in calves (4). As such, γδ T cells are likely to be critical to function in bovine immunity. Although bovine γδ T cells have been suggested to have NK-like or CTL-like activity, only a minor proportion of γδ T cells have been shown to express IFN-γ and/or perforin in response to bacterial, viral, or nonspecific activation (49, 50). However, our studies have confirmed and extended previous findings (9) that T cells expressing the γδ TCR are a major regulatory and suppressive T cell compartment in ruminants.

A large proportion of bovine γδ T cells express WC1, a transmembrane glycoprotein and member of the scavenger receptor cysteine-rich family, which is closely related to CD163. Functional WC1 molecules have so far been identified only in ruminants, pigs, and camelids (51, 52), although there is molecular evidence of the existence of WC1 orthologs in mice and humans (5). Human and murine γδ T cell subsets are tissue specific as illustrated by the distribution of G and D gene segment usage in lymphocytes from various tissues (53, 54). The TCR δ-chain repertoire in cattle has been shown to be highly diverse and greatly expanded to include 56 TRD variable (V) genes, 5 diversity (D) genes, 3 junctional (J) genes, and 1 constant (C) gene (55, 56). This expansion of TRD genes in cattle compared with humans and mice suggests a distinct role for γδ T cells in ruminants (57).

Until now, the induction, phenotype, and function of Tregs in ruminants has not been fully described. However, there is indirect evidence of a regulatory role for γδ T cells in cattle. For example, in vivo depletion of WC1-expressing γδ T cells in cattle after FMDV infection resulted in a shorter period of viremia (5 d) compared with depletion of CD4^+^ (∼6 d) or CD8^+^ T cells (∼7 d) (45). Another study showed that depletion of WC1^+^ γδ T cells resulted in an enhanced Ab response to the model Ag OVA (58). Similarly, enhanced local and systemic bovine respiratory syncytial virus–specific Ab responses were seen post respiratory syncytial virus infection in WC1^+^-depleted calves (59). One major drawback of these studies is that only cells expressing WC1 were depleted. The expression of WC1 in γδ T cells is variable in that 15–60% of all peripheral blood bovine γδ T cells may express WC1, and thus in the aforementioned experiments, not all γδ T cells were depleted from the system. Even though there was likely to be incomplete depletion of all γδ T cells in these in vivo studies, there was evidence that removing these cells could enhance immune responses.

Our data show for the first time, to our knowledge, that up to 15% of bovine γδ T cells express IL-10 ex vivo (Fig. 1). These cells do not express either Foxp3 or IFN-γ, and IL-10 production is not related to WC1 expression. Further inspection of the WC1^+^ population shows that most WC1^+^ IL-10^+^ cells are of the WC1.2 subset (Fig. 1). We and others (9, 40, 60) have unsuccessfully tried to identify bovine CD4^+^ CD25^+^ Foxp3^+^ IL-10^+^ cells with regulatory function, and although the presence of bovine CD4^+^ Foxp3^+^ T cells has been reported (40) (Fig. 1), these cells do not appear to have suppressive capacity. Because γδ T cells represent a major proportion of the lymphocytes in the blood of ruminants, we depleted these cells before stimulation of the remaining cells. By doing this, we were able to increase both polyclonal and Ag-specific proliferation and IFN-γ, IL-2, and IL-4 released by CD4^+^ and CD8^+^ T cells (Fig. 2).

It has been reported that bovine γδ T cells proliferate in culture in the absence of other stimuli (61–63). We now show that up to 15% of these proliferating γδ T cells secrete IL-10 and have regulatory functions. Of the IL-10^+^–expressing γδ T cells, two distinct populations were observed: WC1^+^ and WC1^−^ (Fig. 1J). IL7R and CD45RO have been suggested as alternative markers to identify T cells with regulatory potential (64), and our data show that ∼50% of the IL-10^+^ γδ TCR^+^ cells express IL-7R and most are CD45RO^+^ (Fig. 4D, 4E). In addition, we could not detect expression of Foxp3 on expanding IL-10^+^ γδ T cells. This indicates that the markers suggested so far for identifying Treg populations in humans and mice do not apply to cattle.

Further analysis of the IL-10^+^WC1^+^ population shows only those cells expressing the WC1.2 subgroup were able to proliferate and express IL-10 (Fig. 4F). In contrast, only those cells expressing the WC1.1 subgroup were able to express IFN-γ in response to cytokine or mitogen stimulation. These data suggest that differential expression of specific WC1 genes correlates with bovine γδ T cell function and in the context of Ag as previously suggested (65, 66), and that the serological definition of WC1 subsets does not correlate with function, because all serologically defined WC1 subgroups are able to express proinflammatory and anti-inflammatory cytokines (49, 57, 67).

To determine the signals required to expand IL-10^+^ γδ T cells, we used Abs against several cytokines to try to inhibit this effect (Table I). The presence of IL-10 and TGF-β blocking Abs individually reduced the capacity of γδ T cells to express IL-10 and proliferate, and in combination these two Abs almost completely blocked this effect. The addition of IL-4 and TNF-α blocking Abs did not have a significant effect. Monocytes (CD14^+^) were required for γδ T cell survival, and γδ T cells needed to be in physical contact with CD14^+^ cells. All these data suggest that for γδ T cells to survive, express IL-10, and proliferate, soluble IL-10 and, to a lesser extent, TGF-β are required along with other signals provided by physical contact with CD14^+^ cells.

We were unable to expand IL-10^+^ γδ T cells by culturing with anti-CD3 and anti-CD28 mAbs, indicating that these signals are not required or are insufficient for maintenance of the major Treg compartment in cattle. Murine and human Tregs require engagement of TCR for expansion and function (68); this may also be the case in the bovine system, and more work is required to identify these signals.

Most assays to investigate Treg function rely on the expansion in vitro of the Treg subset in question; therefore, we tested the suppressive activity of in vitro–expanded γδ T cells. Both polyclonal and Ag-specific proliferation of CD4^+^ and CD8^+^ T cells were reduced in the presence of in vitro–expanded γδ T cells (Fig. 5A–D). Similarly, the frequency of IFN-γ^+^ and perforin^+^ CD8^+^ T cells was reduced in the presence of γδ T cells (Fig. 5E–H). Hoek and colleagues (9) reported the inhibitory effect of γδ T cell WC1^+^ subpopulations on polyclonal activation of autologous T cells and observed that with WC1.1^+^ and WC1.2^+^ had suppressive function. However, the authors did not investigate the effect of WC1^−^ γδ T cells or their regulatory function on Ag-specific responses. We did not identify a differential regulatory role between WC1^−^ and WC1^+^ γδ T cell subsets and within the WC1^+^, we observed that only the WC1.2^+^ had suppressive functions; therefore, more work is required to reconcile the observed differences.

For T cells to have regulatory function, these need to obtain the necessary signals from the surrounding environment, including cytokines and DCs. These cells are sometimes called tolerogenic DCs and are a heterogeneous mix of APCs that differ not only with regard to phenotype, differentiation, and maturation status, but also with regard to tolerance-inducing capacity. Although immaturity appears to be a good indicator of DC tolerogenicity, mature DCs may also contribute to the generation and maintenance of Tregs. Various subpopulations of DCs have been shown to maintain Tregs, and these include CD8α^+^, CD4^+^, CD103^+^, CD103^−^, CD11b^+^, CD205^+^, plasmacytoid DCs, and Langerhans cells (extensively reviewed in Ref. 48). The secretion of IL-10, TGF-β, and retinoic acid by DCs has been shown to be necessary for the induction of Tregs (69, 70). We investigated the ability of bovine DCs from three sources, peripheral blood, lung, and afferent lymph draining the skin, to induce IL-10^+^ γδ T cells. Our data show that the major APC populations expressing IL-10 and TGF-β were in peripheral blood, monocytes (MHCII^+^ CD14^+^); in the lung MHCII^+^CD11c^+^SIRPα^+^CD8α^+^ DCs; and in the afferent-lymph MHCII^+^ CD11c^+^SIRPa^−^CD8a^−^ DCs (Figs. 6–8). All of these populations were capable of inducing proliferation of IL-10^+^ γδ T cells with suppressive function. Interestingly, the capacity of CD14^+^ to express large amounts of IL-10 in culture is dependent on the presence of IL-10–expressing γδ T cells (Supplemental Fig. 1), and this may be explained by a positive-feedback mechanism for the production of IL-10 as described in other systems (69, 70). It is possible that CD4^+^ and CD8^+^ T cells may become licensed regulatory cells when in contact with the DCs described earlier; however, we have not yet investigated the role of bovine αβ T cells subsets in immune regulation.

The use of viral vectors to vaccinate against infectious diseases has been investigated for many years. MVA and AdV5 are two of the most commonly used vaccine vectors being tested to date. MVA has been shown to be safe in laboratory animals and target species. However, its efficacy as a vaccine delivery vector has not been consistent. We have previously shown that MVA reduces cell-surface expression of MHC class II, CD40, and CD86 (15), and induces apoptosis in DCs, preventing optimal Ag expression and presentation (71). We hypothesized the consequence of using MVA was that T cell responses were suboptimal not only because of poor Ag presentation and DC death, but also because of the generation of suppressive γδ T cells. Our data show this is, in fact, the case: DCs infected with MVA upregulate the production of IL-10, inducing the proliferation of IL-10^+^ γδ T cells. We confirmed these results by inhibiting IL-10 using blocking Abs and removing γδ T cells from the system (data not shown). There are reports of similar observations in other systems; for example, Kastenmuller and colleagues (72) showed in mice that MVA-induced Tregs selectively limit the number of effector T cells generated whereas preserving the memory response by changing the amount of CD80 and CD86 displayed on the MVA-infected DCs and the availability of IL-2.

Although the role of bovine γδ T cell in immune regulation is unequivocal, it has also been observed that both bovine CD4^+^ and CD8^+^ T cells have suppressive functions in response to staphylococcal enterotoxin (73). Clearly, there are multiple sources of immune-regulating cells in cattle, as there are in humans and mice, and more work is required to identify their role in disease and immune responses and to reconcile apparently opposing data.

Increasing evidence supports the notion that γδ T cells may play an important role in immune regulation in humans and mice (74–76), producing large amounts of IL-10 and suppressing Ag-specific T cell proliferation and activation. Our data demonstrate that the ruminant immune system uses γδ cells as a principal immune-regulatory subset.

## Supplementary Material

Data Supplement
