# Supplementary material for: Bovine γδ T Cells Are a Major Regulatory T Cell Subset
Source: J Immunol. 2014 Jun 2;193(1):208–22. doi: 10.4049/jimmunol.1303398 (PMC4065783; doi:10.4049/jimmunol.1303398)
Supplement: Data Supplement [file supp_193_1_208__index.html]

Bovine γδ T Cells Are a Major Regulatory T Cell Subset — Data Supplement 

# Bovine γδ T Cells Are a Major Regulatory T Cell Subset

## Data Supplement

**Files in this Data Supplement:**

- Supplemental Figure 1 (PDF)
