# Supplementary material for: Bovine γδ T Cells Are a Major Regulatory T Cell Subset
Source: J Immunol. 2014 Jun 2;193(1):208–22. doi: 10.4049/jimmunol.1303398 (PMC4065783; doi:10.4049/jimmunol.1303398)
Supplement: Data Supplement [file 1303398_JI_1303398_Supplemental_Figure_1.pdf]

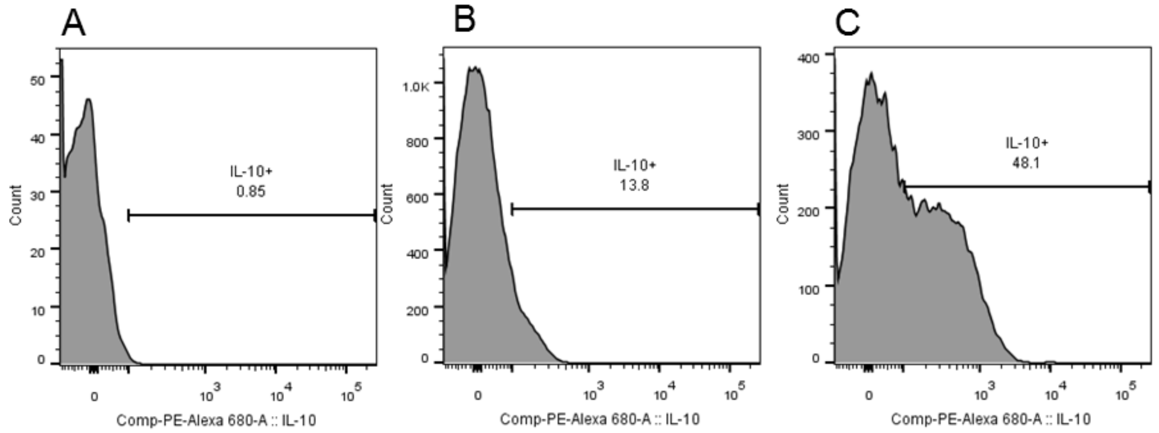

S1. Expression of IL-10 in cultured CD14$^+$ cells. MACS-sorted CD14$^+$ cells were cultured for 3 days in culture media in the absence (B) or presence (C) of autologous $\gamma\delta$ T cells; (A) shows the isotype control. After 2 days in culture brefeldin A was added and the cells incubated overnight. After harvesting, cells were analyzed for the expression of intracellular IL-10. Histograms were gated on live/single events, CD3$^-$ sIg$^-$ MHCII$^+$. Histograms are representative of cells from 3 different animals.
